# Analyses of Bone Regeneration Capacity of Freeze-Dried Bovine Bone and Combined Deproteinized–Demineralized Bovine Bone Particles in Mandibular Defects: The Potential Application of Biological Forms of Bovine-Bone Filler

**DOI:** 10.1055/s-0041-1736291

**Published:** 2021-11-23

**Authors:** David Buntoro Kamadjaja, Handhito Satriyo, Aris Setyawan, Yeni Dian Lesmaya, Jefry Wahyudi Safril, Ni Putu Mira Sumarta, Andra Rizqiawan, Coen Pramono Danudiningrat, Ta To Tran

**Affiliations:** 1 Department of Oral and Maxillofacial Surgery, Faculty of Dental Medicine, Universitas Airlangga, Surabaya, Indonesia; 2 Department of Oral Surgery, Faculty of Dentistry, Van Lang University, Ho Chi Minh, Vietnam

**Keywords:** bovine, deproteinized, demineralized, osteoconductive, osteoinductive, bone filler, innovation

## Abstract

**Objective** This study aimed to evaluate bone regeneration capacity of FDBX granules compared to composite DBBM/DFDBX granules for filling of bone defect in rabbit mandible.

**Material and Methods** Critical size defects were created in 45 rabbits' mandible. The defect in the control group is left untreated, while in other groups the defects were filled with FDBX granules and composite DBBM/DFDBX granules, respectively. Specimens were collected at 2, 4, and 8 weeks for histology and immunohistochemical analyses. Significant difference is set at *p* -value < 0.05.

**Results** The osteoblast-osteoclast quantification, osteoblast expression of Runx2, alkaline phosphatase, collagen-I, and osteocalcin, and osteoclast expression of receptor activator of NF-kB ligand (RANKL) and osteoprotegerin (OPG) in FDBX groups were statistically comparable ( *p* > 0.05) with the composite group, while OPG/RANKL ratio, bone healing scores, and trabecular area were significantly higher ( *p* < 0.05) in the composite compared to FDBX group.

**Conclusion** Composite DBBM/DFDBX granules, within the limitation of this study, has better bone forming capacity than FDBX granules for filling of bone defects in the mandible.

## Introduction

The demand for bone graft substitutes in implant treatment has increased a great deal for the past decades. Human bone-derived particles such as freeze-dried bone allograft (FDBA) have been used as osteoconductive particles while *demineralized FDBA* (DFDBA) has been applied due to its osteoinductive properties. 1 However, human bone products processed by tissue bank has typical problem of lack in donor supply. Furthermore, allogenic bone grafts have been associated with risk of disease transmission from donors to recipients. 2 Consequently, bone substitutes materials have been increasingly popular and, in this respect, *deproteinized bovine bone mineral* (DBBM) particles is the most widely used bone substitute in implant and periodontal surgeries. 3

The DBBM particle is purely inorganic that is considered osteoconductive and exhibits extremely slow degradation which may reduce the bone regeneration capacity and bone-implant contact area. 4 5 Limitation of DBBM particle has made way to the utilization of other forms of bovine bone particles, that is, freeze-dried bovine bone particles (FDBX) and demineralized freeze-dried bovine bone particle (DFDBX) recapitulating the processing in allograft production. 6 Despite concerns over the potential xenogeneic response upon its application, these organic forms of xenograft material is proven to be nontoxic and nonimmunogenic in *in vitro* and *in vivo* studies. 7 8 9 Much similar to human FDBA, the bovine FDBX retains inorganic and organic components which make it osteoconductive and have some amount of osteogenic growth factors. Furthermore, it undergoes complete degradation to support bone regeneration. 10 On the other hand, DFDBX is purely organic believed to have osteoinductive properties as it release various osteogenic growth factors such as bone morphogenetic proteins (BMPs) and transforming growth factor (TGF)-β. However, DFDBX particle has poor structural strength as it is devoid of inorganic components. 11

Taking into consideration the different biological and mechanical properties of the three types of bovine bone particles, we assume that FDBX particles should have bone healing capacity comparable with that of combined DBBM-DFDBX particles when utilized for filling of bone defect. This animal study aims to compare the osteogenic processes and bone forming capacity of mineralized FDBX and combined DBBM-DFDBX particles in a nonspacemaking, critical size mandibular bone defect.

## Material and Methods

**Fig. 1:**
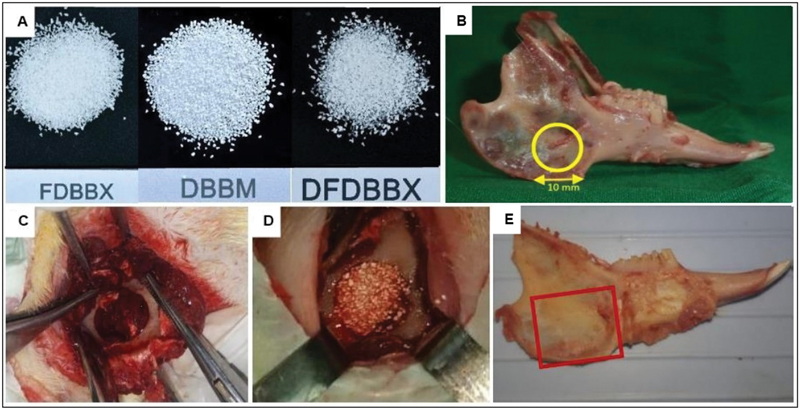
Bone grafting of critical size defect in rabbit mandible. ( **A** ) the freeze-dried bovine bone xenograft (FDBX), deproteinized bovine bone mineral (DBBM), and demineralized bovine bone xenograft (DFDBX) particle of 150–300 μm; ( **B** ) location of the critical-size-defect at the ascending ramus of the rabbit mandible; ( **C** ) the bicortical, 10-mm, round-shaped defect created in the ramus of rabbit mandible prior to grafting and ( **D** ) after grafting with composite particle; ( **E** ) the picture of rabbit mandible after euthanasia showing the margins of specimen collection at the ramus bone (rectangular line delineating margins of resection).

This posttest control group design study uses New Zealand white rabbit as the experiment subject. The animal research procedure was approved by research ethics committee, Faculty of Dental Medicine, Universitas Airlangga, Surabaya, Indonesia (022/HRECC.FODM/I/2019). The inclusion criteria were 6-month-old, male rabbit weighing 3 to 3.5 kg. Animal who suffered from wound infection or died before the experiment termination were excluded from this study. The experimental research was conducted in the Animal Research Laboratory, Stem Cells Research and Development Center, Universitas Airlangga, Surabaya, Indonesia. Graft material used in this study is lyophilized (freeze-dried) bovine bone xenograft (FDBX), DFDBX, and DBBM with particle size of 150 to 300 μm ( Fig. 1A ). They were all processed by Tissue Bank, Dr. Soetomo Hospital, Surabaya, Indonesia.

Critical-size defect was created in the ascending ramus of mandible ( Fig. 1B ) of 45 rabbits which were randomly divided into three groups. In the FDBX group the bone defects were filled with FDBX particles, in the composite group with mixture of DBBM and DFDBX particles at 1:1 weight ratio while in the Control group the defects were left unfilled. The surgery was blindly performed by three operators. Two, 4, and 8 weeks after bone grafting surgery five rabbits from each group were sacrificed to collect specimens for histology and immunohistochemistry analysis.

### Processing of Bovine Bone Particles

The bovine femoral bone was washed in pressurized lavage with distilled water to remove blood and marrow. Pasteurization was done in water bath shaker at 60°C for 5 hours followed by dissection to remove residual fat and soft tissue. The bone was cut into smaller pieces and then underwent second cleaning, started with distilled water followed by hydrogen peroxide solution until the bone turned white. Following washing with distilled water, the bone was immersed in hexane solution to remove the residual fat from marrow cavity. The bone chips were then kept in deep freezer for 24 hours followed by dry heating until the water content was less than 10%. The bone chips were placed in sterile vials and kept in sealed plastic pouch for sterilization process.

In order to have demineralized graft particles (DFDBX) a demineralization procedure is performed by immersing the bone chips in 0.1% hydrogen chloride solution until soft consistency was achieved, thereafter the bone chips were again washed with normal saline solution until the pH of 7 was reached. The soft bone pieces were then cut up into smaller chips, ground, and filtered to obtain particle size 150 to 300 μm. The freeze-drying procedure was performed as previously described above. Finally, particles were placed in sterile vials and kept in double-layer plastic packaging prior to sterilization by gamma ray irradiation.

### Surgical Procedure

Critical size defect model used in this study was a bicortical, 10-mm round-shaped hole created with trephine bur at the angle of rabbit's mandible as shown above ( Fig. 1B ). The rabbits were anesthetized by intramuscular injection of ketamine HCl at a dose of 20 mg per kg body weight followed with xylazine 3 mg per 3 kg body weight. After hair shaving and disinfection over the right mandible, skin incision and dissection of the subcutaneous tissue were done until inferior border of the right mandible was reached. Under copious saline irrigation a round shape defect was created with trephine bur as described above ( Fig. 1C ). The drilling was started from the buccal cortical bone further advanced medially and stopped once the lingual cortical plate was breached in order to keep the lingual periosteum intact. The defects in the treatment groups were randomly filled up with FDBX or composite (mixed DBBM/DFDBX) particles ( Fig. 1D ), whereas in the control group, the defects were not filled with any bone graft materials. The buccal periosteum was then repositioned and stitched firmly to the lingual periosteum to support the stability of the graft particles in the bony defects. The overlying tissue was closed layer-by-layer with absorbable 4-0 suture material and the skin with silk 3-0 suture.

At the end of second, fourth, and eighth week post-graft implantation, five rabbits from each group were sacrificed to collect specimens for tissue analysis. The procedure of specimen collection was conducted as follows. The rabbits were euthanized with xylazine injection followed by exsanguination. The animal's death was confirmed with cessation of circulation. The skin overlying the mandible is dissected and the mandible removed from its articulation with the skull. Bicortical resection of the respective alveolar bone was carried out along with 1 to 2 mm surrounding bone ( Fig. 1E ). The specimens were soaked in 10% buffered formaldehyde solution for at least 3 days before further processing procedure.

### Histology Examination

The specimens were decalcified with 10% ethylenediaminetetraacetic acid until decalcification completion was confirmed with prick test. The decalcified specimens were embedded into paraffin block, slicing done at 4 μm thick, which then deparaffinized with xylene, rehydrated in 100% alcohol, and washed in distilled water. The slides were then stained with hematoxylin and eosin. 12

The findings from histology examination were used for quantitative assessment of osteoclast and osteoblast, semiquantitative assessment of bone healing, and histomorphometry of trabecular area. The osteoblasts and osteoclasts assessment were randomly performed around the graft throughout the defect over 10 observation fields. Osteoblasts and osteoclasts were designated as hexagonal-shaped cells and multinucleated cells, respectively. The cell counting was done blindly by two randomly assigned examiners using digital-aid cell counting.

The semiquantitative assessment of bone healing at 4 and 8 weeks were performed with the use of modified histological scoring system. 13 Briefly, the scoring system ranging from “1” to “4” were designated to the proportion of various callus tissue which were fibrous tissue, hyaline cartilage, woven bone, and trabecular bone found in a sample. The distribution of scores in each group at one time point was summed and divided by 5 to obtain mean score of bone healing in each group. Finally, histomorphometry assessment of bone formation was conducted by measuring the trabecular area in the defect at 4 and 8 weeks. All of the examination was performed with light microscope at magnification of ×40 and ×100, while the cell counting and measurements were done manually by two blinded persons aided by image software ImageRaster version 3.

### Immunohistochemistry Examination

Samples for immunohistochemical staining were incubated in 3% peroxide acid for 30 minutes to block endogenous peroxidase, soaked in 0.025% trypsin-phosphate buffer saline for 6 minutes, and finally washed with distilled water three times for 2 minutes. Samples were stained for 30 minutes with mouse monoclonal anti-rabbit Runx2, receptor activator of NF-kB ligand (RANKL), and osteoprotegerin (OPG) antibodies (Santa Cruz Biotechnology Inc., USA), mouse monoclonal anti-rabbit osteocalcin, collagen-I, and alkaline phosphatase (ALP) (Novus Biological, USA). The samples were then immersed in secondary antibody *Polytek HRP Anti-Rabbit Polymerized* (Syntec Laboratories, USA) for 30 minutes at room temperature. Finally, the samples were soaked in substrate DAB *Chromogen* ACB002 mixed with DAB *Substrate High Contrast* ACU005 for 5 minutes. After washing three times in phosphate-buffered saline, *bluing reagent* (BRT 125) was applied for 5 minutes which was directly washed in distilled water. Finally, the slides were cleaned with *xylene* and followed by *mounting* for examination with light microscope (BX-41 model, Olympus, Japan) using digital camera (DP-70 model, Olympus). The positive intracellular protein expression of collagen-I, ALP, osteocalcin, RANKL, and OPG were indicated by brown-stained cytoplasm of osteoblast, except for Runx2 expression which was shown by brown staining of osteoblast's nuclei.

The data obtained from this method was the total number of osteoblast positively stained with the respective antibodies. The counting was done manually on 20 visual fields in each sample performed by two randomly assigned examiners. The data was presented as mean number of positively stained cells from five replications. The data of osteoblastic expressions of Runx2, collagen-I, ALP, and osteocalcin were used to represent the osteogenic differentiation and osteoblastic maturation processes, while osteoblast expression of RANKL and OPG evaluate the process of osteoclastic differentiation during bone healing.

### Statistical Analysis

Statistical analysis was performed with software package SPSS version 17 (IBM Inc.). Data from each experiment group was statistically analyzed with the assumption of homogeneity of variances and normal distribution of errors being tested for the variables examined. One-way analysis of variance followed by Tukey's Honest Significant Difference multiple comparison test and Kruskal–Wallis followed by Dunn's post-test were used for quantitative and semiquantitative data, respectively, with significance set at *p* -value *<* 0.05.

## Results

### Microscopy of Bone Defect Healing at 2 and 4 Weeks Post-Bone Grafting

**Fig. 2:**
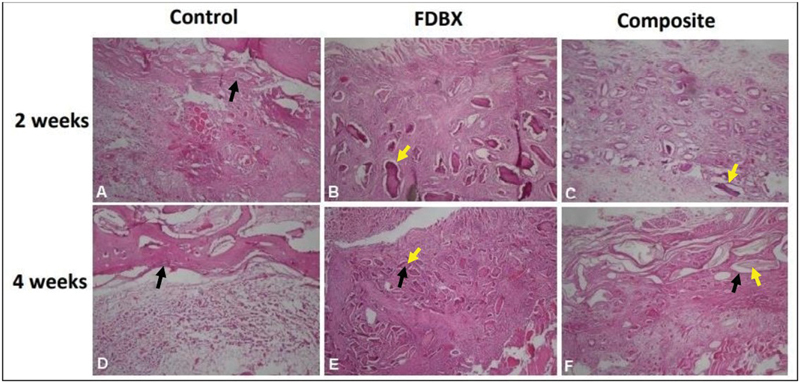
Histology of bone defect healing 2 and 4 weeks after bone grafting. ( **A** , **D** ) Control groups showing mineralized callus adjacent to bone edges of the defects. ( **B** , **E** ) Freeze-dried bovine bone xenograft (FDBX) group at 2 weeks showed grafts materials surrounded by dense connective tissue which were replaced by marked bone formation at 4 weeks. ( **C** , **F** ) Composite groups at 2 weeks showed graft materials in the dense mesenchymal tissue, at 4 weeks woven bone formed surrounding the residual graft materials (yellow arrow pointing to residual graft material, black arrows to newly formed bone, hematoxylin and eosin [H&E] staining, ×40 magnification).

The microscopy of histology at 2 weeks showed that the defects in the control group were filled with dense connective tissue and hyaline cartilaginous tissue, whereas in the FDBX and composite groups, graft materials in central zones were surrounded by dense connective tissue across the defect. At 4 weeks postimplantation, the control group showed bone trabeculae along the bone edges with dense connective tissue in the central zone of the defect. The central zone in the FDBX group showed residual graft materials which were gradually replaced by woven bone. The composite group, on the other hand, revealed that the residual graft materials were not replaced by bone tissue, rather, they were surrounded by mineralized tissue in the form of woven bone ( Fig. 2 ).

### Quantitative Assessment of Osteoclast and Osteoblast

**Fig. 3:**
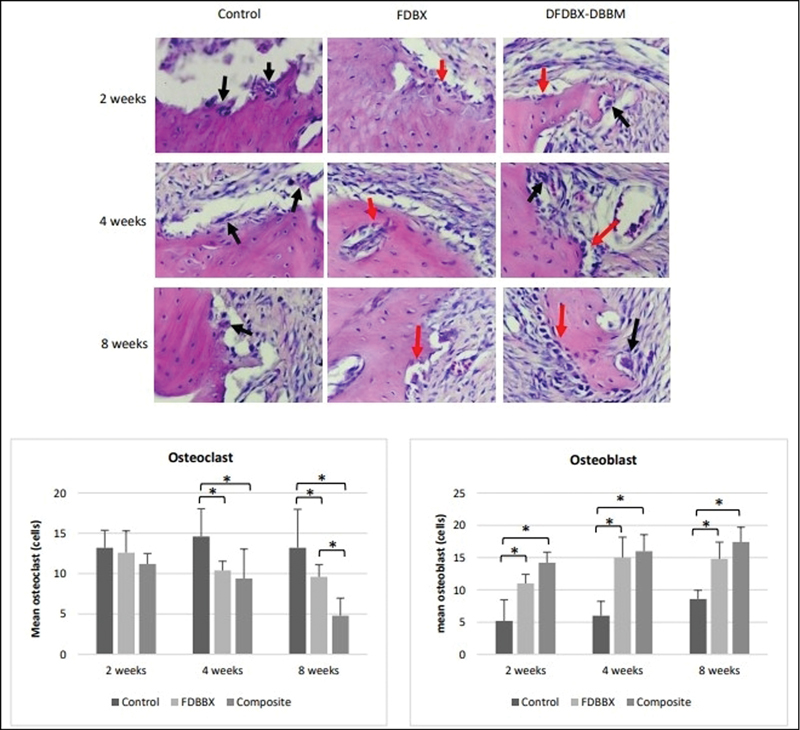
Quantitative assessment of osteoclast and osteoblast at 2, 4, and 8 weeks (the arrows in black point to osteoclast, red to osteoblast, mature bone is either bone edge [control group] or residual graft materials [in grafted groups], hematoxylin and eosin [H&E] staining, ×400 magnification). The mean number of osteoclast in the control group are consistently higher than the freeze-dried bovine bone xenograft (FDBX) and composite group, while the mean number of osteoblast in the FDBX and composite groups are significantly higher than in the control group ( *p* < 0.05). There exists an interdependent relationship between the number of osteoblast and osteoclast at 2, 4, and 8 weeks. While osteoblast count is consistently increased and osteoclast decreased from early to later stage of healing, the osteoclast count in the control groups is seen to be consistently high at all time points.

The data from histology quantitative assessment show that the mean number of osteoblast in the FDBX and composite group was statistically comparable to each other but they were both significantly higher than the control group at 2, 4, and 8 weeks. The mean number of osteoclast in the control group is, on the other hand, significantly higher than the FDBX and composite group ( *p* < 0.05). It was also shown that there were inverse relationships between osteoblast and osteoclast count in all the groups at 2, 4, and 8 weeks. While mean osteoblast number increase and osteoclast decrease in majority of groups, the osteoclast number in the control groups was seen to increase with time ( Fig. 3 ).

### Osteoblastic Differentiation and Maturation

**Fig. 4:**
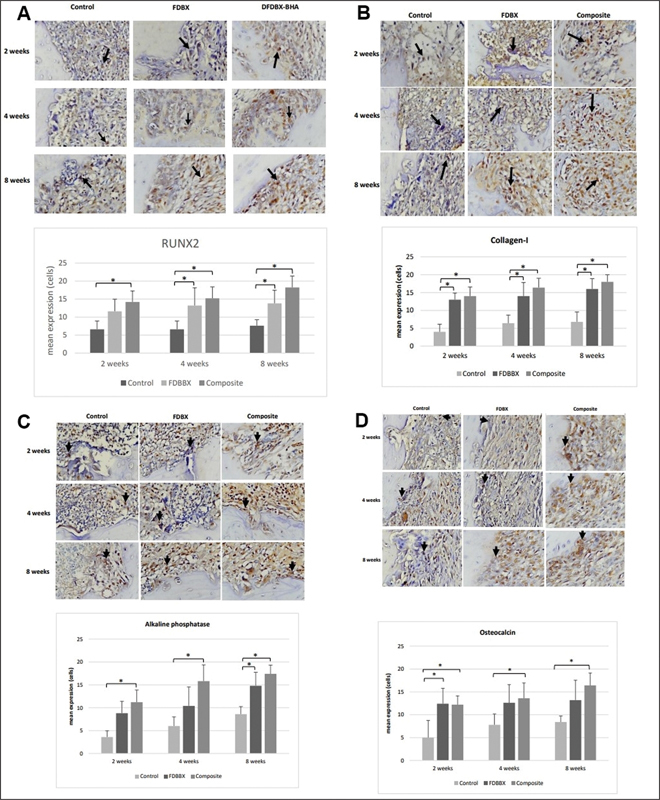
Expression of osteoblastic differentiation and maturation markers. It is shown that mean expression of ( **A** ) RUNX2, ( **B** ) collagen-type I, ( **C** ) alkaline phosphatase, and ( **D** ) osteocalcin constantly increase until 8 weeks in all groups. The mean expression of those osteoblastic markers in the freeze-dried bovine bone xenograft (FDBX) and composite group is significantly higher ( *p* = 0.001) than the control group in all time points; however, no statistical difference ( *p* < 0.05) is found in all the above markers between the FDBX and composite groups throughout the observation periods.

The result of immunohistochemistry analysis of osteoblastic differentiation markers shows that the mean expression of RUNX2, ALP, collagen-I, and osteocalcin were still increasing until 8 weeks in all groups. It was also shown that all the osteoblastic markers in the FDBX and composite groups were significantly higher ( *p* = 0.001) than the control group at the three time points of observation. While all osteoblastic differentiation and maturation markers in the composite group were consistently higher than those in the FDBX group at all time points there was no significant difference ( *p* < 0.05) between the two groups ( Fig. 4 ).

### Osteoclastic Differentiation

**Fig. 5:**
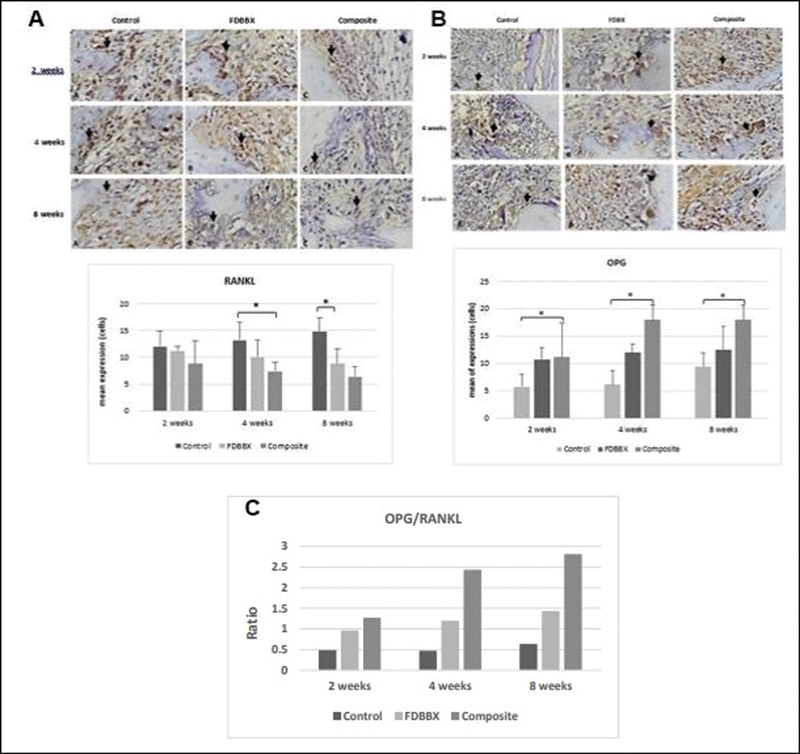
Expression of osteoclastic differentiation markers. ( **A** ) The mean expression of osteoclast RANKL in freeze-dried bovine bone xenograft (FDBX) and composite groups decreases from 2 through 8 weeks of healing as opposed to that in the control groups which show gradual increase with time. The mean expression of osteoclastic RANKL in the control groups is significantly higher ( *p* < 0.05) than the FDBX and composite group at 4 and 8 weeks of healing. ( **B** ) The mean osteoblast expression of osteoprotegerin (OPG) protein increases gradually through the observation periods, no significant difference is observed in OPG expressions between the composite and FDBX groups ( *p* > 0.05) during the same periods of healing. ( **C** ) The OPG/RANKL ratio in the composite groups is much higher, > twofold those in the FDBX group specifically at 4 and 8 weeks indicating more osteoblastic activities in the composite group.

The immunostaining results exhibit that mean osteoblastic RANKL expression in the control group increases until 8 weeks of healing as opposed to those in the FDBX and composite groups which gradually decrease with time. The mean expression of RANKL in the control group was found significantly higher than the FDBX and composite group at all time points. The mean expression of OPG tends to increase until 8 weeks, except for the FDBX group which exhibits relatively constant expression. While the osteoblast expression of OPG in the composite group was higher than the FDBX group specifically in the last 4 weeks of healing, no significant difference was observed ( *p* > 0.05) between the composite and FDBX groups ( Fig. 5 ). The data also shows that OPG/RANKL expression ratio in the composite group was consistently the highest among all groups and at all time points.

### Semiquantitative Assessment of Bone Healing

**Fig. 6:**
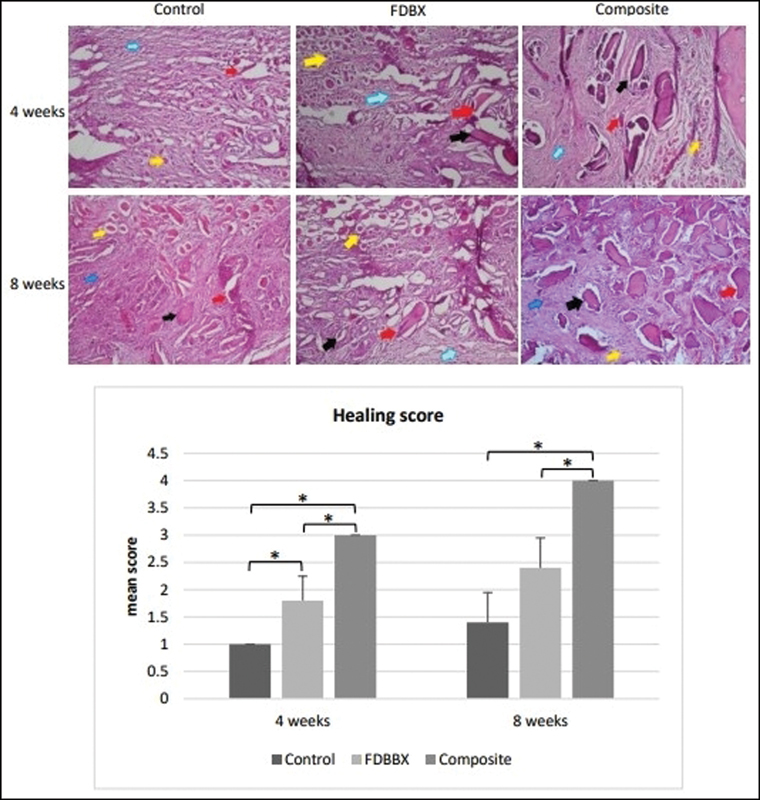
Semiquantitative assessment of bone healing in the control, freeze-dried bovine bone xenograft (FDBX), and composite group at 4 and 8 weeks of healing. The mean score of bone healing in the composite and FDBX groups is consistently higher than the control groups at 4 and 8 weeks; furthermore, the healing scores in the composite groups are found to be significantly higher ( *p* < 0.05) compared to the FDBX groups at those two time points (note: blue arrows pointing to connective tissue, yellow to hyaline cartilage, red to woven bone, black to trabecular bone, hematoxylin and eosin [H&E] staining with ×100 magnification).

The histology comparative assessment of bone healing shows a clearly distinct level of tissue maturation in the control group compared to the FDBX and composite group at 4 weeks of healing. Fibrous tissue and hyaline cartilage were the major tissues in the control group with small amount of bone tissue at the periphery of the defect, while woven bone were more evident in the FDBX and composite group. At 8 weeks the bone tissue in the control and FDBX group was more obvious yet still much less compared with the composite group. Semiquantitative assessment of bone healing showed that the bone healing scores in the composite and FDBX groups were significantly higher than the control group at 4 and 8 weeks. In addition, the healing scores in the composite group were significantly higher ( *p* < 0.05) compared to the FDBX group at all time points ( Fig. 6 ).

### Assessment of Trabecular Area (Histomorphometry)

**Fig. 7:**
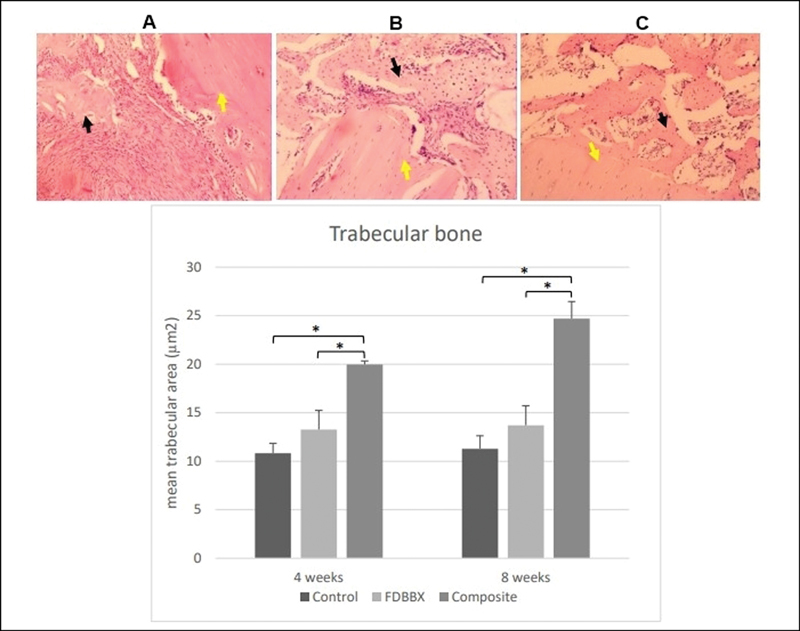
Assessment of trabecular area at 4 and 8 weeks of healing. ( *Top* ) Histology of bone healing at 8 weeks showing woven and trabecular area seen in ( **A** ) the control and ( **B** ) freeze-dried bovine bone xenograft (FDBX) groups, as opposed to interconnecting trabecular area incorporating with the host bone in ( **C** ) the composite groups (black arrows pointing to trabecular area, yellow arrows indicating host bone, hematoxylin and eosin [H&E] staining, ×100 magnification). ( *Bottom* ) The mean trabecular areas in the composite groups are significantly higher ( *p* < 0.05) than the FDBX and control groups at 4 and 8 weeks. The trabecular area in the composite groups increases from 4 to 8 weeks; whereas those in the control and FDBX group are found to remain constant with time.

The comparative histological assessment of healing at low magnification showed distinct streaky pattern of bony tissue formation in the control and FDBX group compared to large, interconnecting trabecular area seen in the composite group at 4 weeks and specifically at 8 weeks. The result of histomorphometry revealed that the mean trabecular area in the composite group increased from 4 to 8 weeks; whereas those in the control and FDBX group remained constant with time. The mean trabecular areas in the composite group were significantly higher ( *p* < 0.05) than the FDBX and control groups at all time points ( Fig. 7 ).

## Discussion

Autogenous or allogeneic bone particulate remains the gold standard bone graft for alveolar bone augmentation associated with dental implant treatment. Limitation in source and availability of these bone grafts have led to the utilization of xenogeneic bovine bone because of its similarity to human bone in terms of porosities, biological, and physical properties. The most widely used bovine bone substitutes were DBBM particles which has good osteoconductive properties and high mechanical stability. 14 However, it was associated with very slow degradation that might lower its bone regeneration capacity. 4 In order to overcome DBBM limitation, mineralized FDBX and DFDBXs were developed recapitulating the concept and processing of human FDBA and DFDBA. 15 Despite the concern over the potential xenogeneic response upon its clinical application, this organic forms of xenograft material did not show abnormal immune response in few *in vitro* 7 16 17 18 and *in vivo* studies 7 9 19 as well as in a clinical study. 8

The FDBX particles were composed of organic and inorganic structures in the form of mineralized extracellular matrix which serve as osteoconductor and osteoinductor. The chemical removal of fat and tissue as well as lyophilization procedures eliminate the antigenicity and pathogenicity of FDBX particle; however, such treatment potentially lower the osteogenic growth factors concentration thus reducing the osteoinductive capability of this particle. 10

The demineralized bovine bone, or DFDBX, particles were processed with acid decalcification which eliminates the mineral phase while preserving the organic components. Much similar to DFDBA, it was known to retain varying concentration of BMPs and TGF-β, the two most important osteoinductive growth factors. 11 However, demineralized bone particles have limited osteoconductive properties and, practically, have no mechanical strength. 20

Based on the above description, we hypothesized that mineralized FDBX particles and combined DBBM/DFDBX particles should have comparable bone regeneration capacity for grafting of bone defects. We evaluate the cellular and biomolecular events in response to grafting, the quality of bone healing, and the quantity of bone formation at various bone regeneration and remodeling stages.

The graft materials, clearly seen either in the FDBX or composite groups in 2 weeks, presented different histological features in 4 weeks of healing. The FDBX graft materials were replaced with woven bone; however, the composite graft materials remained and were surrounded by mineralized tissue ( Fig. 2 ). There is a principle difference in bone healing events between mineralized bone graft and hydroxyapatite (HA) crystalline particles upon implantation. Mineralized graft undergo osteoclastic bone resorption followed by osteoblastic bone formation, the process referred to as “creeping substitution” while the amount of residual HA in DBBM phase of composite graft was more stable due to higher resistance to osteoclastic resorption. 21 The finding in this study was consistent with an experiment in rabbit model showing that residual material in defects filled with DBBM was significantly higher than that observed in defects filled with biphasic calcium phosphate, indicating that DBBM resorption is very slow. 22

Quantitative assessment of osteoclast and osteoblast in this study were to evaluate the bone formation and remodeling events in the defects regenerated with different types of bovine bone particles. The consistently higher osteoclast number in the control group ( Fig. 3A ) reflects active resorption activities throughout the observed time points. This finding was in accordance with the result of a study of fracture sheep model that exhibited high osteoclasts number and density at various stages of fracture healing. At the early stage, osteoclasts were active and absorbed the mineralized endosteal bone to recanalize the medullary cavity and restore vascularity. At later stage, the number of osteoclasts was still high as it was responsible for the absorption of woven callus to form lamellar bone. 23 The early-onset osteoblastic bone formation in the grafted groups ( Fig. 3 ) suppressed osteoclast activities through a modulative osteoblastic expression of OPG and RANKL. 24

Significant increase in osteoblast number was noted in the FDBX and composite groups; however, there were no significant difference documented between the two groups. Both mineralized FDBX and composite DBBM/DFDBX graft particles were considered to have some amount of osteogenic growth factors contained within the extracellular matrix. The difference was in the way by which growth factors were released from the graft matrix. While DFDBX provide more ready-to-use molecules upon macrophages-mediated matrix degradation, the growth factors in FDBX were embedded in mineralized matrix which need more time to be utilized by the tissue. 25

The biomolecular events in osteoblastic differentiation and maturation in the defect were evaluated by immunohistochemistry. Significant increase in the expressions of Runx2, collagen-I, ALP, and osteocalcin documented in the FDBX and composite group were consistent with the significant increase in osteoblast number in both the grafted groups. This increase in these markers may be associated with increased osteoblastic differentiation of mesenchymal stem cells (MSCs) and osteoprogenitor cells populating the defects. These undifferentiated cells were recruited either from circulation, endothelium, or from the surrounding bone marrow by growth factors such as BMPs or BMPs released by the organic matrix of the bone granules. 26

Apart from chemokine effect, the release of BMP2 also increased RUNX2 transcription activity, which induces gene expression related to osteoblast differentiation and improves bone formation. Additionally, TGF-β promotes osteoprogenitor proliferation, early differentiation, and relation between osteoblastic lineage through MAPK and Smad2/3 selective pathway leading to induction of collagen-1 expression. 27 *In vitro* study of MSC osteogenic differentiation reveals maximum ALP expression level until day 14, followed by increase in osteocalcin and osteopontin expression leading to calcium-phosphate deposition. 28

This study also reveals that osteoclast number was interdependently related to osteoblast number in all the groups. The high OPG/RANK expression ratio, specifically in the composite group, at 4 and 8 weeks indicate that osteoblastic bone formation was the dominating event during the remodeling phase in this group. This finding may be associated with osteoblast-osteoclast interaction which occur during bone healing and bone remodeling process. Many studies suggested that osteoblast modulate monocyte-to-osteoclast differentiation through OPG and RANKL expressions. 29 In the RANKL/RANK/OPG pathway, RANKL binds to RANK as its receptor and eventually leads to osteoclast precursor maturation. OPG was known as a decoy receptor for RANKL which prevents RANKL-RANK binding and the following reactions. 30

The bone healing score results in this study were found to be linear with that of histomorphometry whereby the composite group showing higher amount of bone than the FDBX group. This result was supported by one study comparing mineralized cancellous allograft material to a 1:1 weight ratio combination of DFDBA and deproteinized mineralized bovine bone in bilateral sinus grafts and concluded that resorption and replacement by new bone occurred more rapidly in the mineralized cancellous allograft material but that both groups resulted in successful new bone formation. 31 Similar study evaluating the effect of a combination of demineralized bone matrix (DBM) and HA compared with HA alone on osteogenesis *in vitro* and *in vivo* concluded that DBM/HA putty showed better osteoinductivity and conductivity over HA alone. 32

Although FDBX was considered as having some level of osteoinductivity, composite DBBM/DFDBX granules obviously had higher osteogenic induction capacity. In addition to biologic activities through the release of BMPs from DFDBX granules, the DBBM granules also had osteoinductive effect but through a different mechanism from the BMP pathway. HA substrate increased ALP, collagen-I, and osteocalcin gene expressions in uncommitted C3H10T1/2 culture without upregulation of core binding factor 1 (Cbfa1, Runx2). 33 34 As such, strategy of combining DBBM and DFDBX particles not only would increase osteoconductivity but also osteoinductive properties through biphasic osteoblastic differentiation effects.

From the result of this study it may be concluded that, within the limitation of this study, combined DFDBX-DBBM granules had better bone regeneration capacity than mineralized freeze-dried bovine bone (FDBX) granules for filling of bone defect. This might be due to better osteoconductivity and osteoinductivity together with higher mechanical stability. Further study is required to validate bone regenerative efficacy in larger defects and with various ratios combination.
